# Access to HIV prevention services in East African cross‐border areas: a 2016‐2017 cross‐sectional bio‐behavioural study

**DOI:** 10.1002/jia2.25523

**Published:** 2020-06-30

**Authors:** Arti V Virkud, Peter Arimi, Freddie Ssengooba, Grace E Mulholland, Michael E Herce, Milissa Markiewicz, Sharon Weir, Jessie K Edwards

**Affiliations:** ^1^ Department of Epidemiology University of North Carolina at Chapel Hill Chapel Hill NC USA; ^2^ U.S. Agency for International Development Kenya/East Africa Regional Mission Nairobi Kenya; ^3^ College of Health Sciences School of Public Health Makerere University Kampala Uganda; ^4^ Department of Medicine Division of Infectious Diseases University of North Carolina at Chapel Hill Chapel Hill NC USA; ^5^ MEASURE Evaluation Carolina Population Center University of North Carolina at Chapel Hill Chapel Hill NC USA

**Keywords:** HIV, Africa, Eastern, border crossing, condoms, female, vulnerable populations

## Abstract

**Introduction:**

East African cross‐border areas are visited by mobile and vulnerable populations, such as men, female sex workers, men who have sex with men, truck drivers, fisher folks and young women. These groups may not benefit from traditional HIV prevention interventions available at the health facilities where they live, but may benefit from services offered at public venues identified as places where people meet new sexual partners (e.g. bars, nightclubs, transportation hubs and guest houses). The goal of this analysis was to estimate availability, access and uptake of prevention services by populations who visit these venues.

**Methods:**

We collected cross‐sectional data using the Priorities for Local AIDS Control Efforts sampling method at cross‐border locations near or along the land and lake borders of Kenya, Rwanda, Tanzania and Uganda from June 2016–February 2017. This bio‐behavioural survey captured information from a probability sample of 11,428 individuals at 833 venues across all areas. Data were weighted using survey sampling weights and analysed using methods to account for the complex sampling design.

**Results:**

Among the 85.6% of persons who had access to condoms, 60.5% did not use a condom at their last anal or vaginal sexual encounter. Venues visited by high percentages of persons living with HIV were not more likely than other venues to offer condoms. In 12 of the 22 cross‐border areas, male or female condoms were available at less than 33% of the venues visited by persons having difficulty accessing condoms. In 17 of the 22 cross‐border areas, education outreach visits in the preceding six months occurred at less than 50% of the venues where participants had low effective use of condoms.

**Conclusions:**

Individuals visiting venues in cross‐border areas report poor access to and low effective use of condoms and other prevention services. Availability of HIV prevention services differed by venue and population type and cross‐border area, suggesting opportunities for more granular targeting of HIV prevention interventions and transnational coordination of HIV programming.

## 1 INTRODUCTION

Achieving the UNAIDS 2030 goals to reduce new HIV infections to 200,000 per year will require optimization of HIV prevention and testing [1]. While HIV treatment has been crucial in reducing the number of new infections, combination HIV prevention to reduce transmission risk is still necessary to meet these goals [2]. Primary HIV prevention interventions include routine HIV testing services (HTS), provision of condoms and sexual lubricants, pre‐exposure prophylaxis (PrEP), post‐exposure prophylaxis (PEP), voluntary medical male circumcision, sexually transmitted infection (STI) screening and treatment, and needle and syringe programmes. These primary prevention interventions have all been shown to reduce new infections [1, 3, 4, 5, 6].

East African cross‐border areas are important mixing grounds for populations at risk of acquiring HIV and may be underserved by national prevention efforts. Social venues, like bars and nightclubs, in these areas are visited by a diverse population often looking to meet new sexual and needle‐sharing partners, and are exposed to a unique blend of national and local HIV prevention programming. These areas are visited by traditionally defined key populations, such as female sex workers, men who have sex with men and persons who inject drugs, as well as other populations at elevated risk for HIV, such as truck drivers, fisher folk (persons who self‐identify as engaged in fishing industry) and young women [7, 8, 9, 10]. The presence of these key and priority populations make cross‐border areas important focal points for HIV prevention efforts [11, 12].

While national HIV strategic plans across East African countries recommend similar prevention interventions, the prioritization of key populations differs across national plans [7, 8, 9, 13]. Key and mobile populations visiting cross‐border venues may not be reached by recommended facility‐based prevention services near where they reside [14, 15]. Better specifying gaps in HIV prevention programming using a cascade analysis and geographically targeting effective services to the places where mobile and key populations meet and socialize can help ensure that these populations are not neglected in regional cross‐border HIV prevention efforts [16].

The availability and effective use of HIV prevention services at East African cross‐border areas is not well understood. A deeper understanding of how these services are distributed and where gaps exist can help drive improvement of existing HIV prevention programmes and the introduction of future interventions. In this paper, we describe the distribution and uptake of primary HIV prevention services, specifically condom availability, at venues identified as places where people meet new sexual partners in cross‐border areas. We highlight the extent to which HIV prevention service availability aligns with the presence of high‐risk behaviours and HIV prevalence. We also examine gaps in prevention access and utilization through the lens of a programmatically relevant “prevention cascade” framework [17, 18, 19, 20, 21] and map disparities between services nominally offered at cross‐border venues and their availability as reported by venue patrons.

## 2 METHODS

### 2.1 Study population

We examine the distribution of prevention services in East Africa cross‐border areas using data from the Cross‐Border Integrated Health Study (CBIHS), described in detail elsewhere [15]. Briefly, the CBIHS collected data describing the health status and behaviours of populations living in and/or travelling through 14 cross‐border areas, representing 22 unique locations in the East African countries of Kenya, Rwanda, Tanzania and Uganda between June 2016 and February 2017. The selected cross‐border areas were chosen based on high level of cross‐border traffic and/or trade and sizeable populations. Of the selected areas, eight surrounded international border posts on major transport corridors, and six were situated around fishing villages on Lake Victoria that serve as points of international commerce.

### 2.2 Study data collection

In each selected area, the Priorities for Local AIDS Control Efforts (PLACE) method was used to collect information on venues where people socialize and meet new sexual partners and to collect health behaviour and outcome data for people at these venues [14, 22, 23, 24, 25]. The PLACE method, explained in Data S1, aims to help local officials prioritize and allocate resources by identifying and characterizing populations that may benefit from HIV prevention and treatment services and venues where these populations can be reached.

### 2.3 Ethics

Study protocol (IRB number 15‐3234) and activities were reviewed and approved by the Institutional Review Board at the University of North Carolina in Chapel Hill; Makerere University Higher Degrees, Research, and Uganda National Council of Science and Technology; the Kenya Medical Research Institute Ethics Review Committee; the National Institute for Medical Research in Tanzania; and the Rwanda National Ethics Committee. All participants in the bio‐behavioural survey provided written informed consent.

### 2.4 Variable coding

Characteristics of the venues were defined by responses from informants at the venue, hereafter: “informants.” Prevention services examined include distribution of free condoms (male and female), free sexual lubricants, condoms for sale, availability of HIV testing, safer sex education by outreach workers, availability of needle exchange programmes, availability of male circumcision programmes and visits by outreach workers, sex worker peer educators, men who have sex with men peer educators, and/or mobile HIV‐care clinics, as reported by informants. Education outreach was defined as present if informants reported the presence of safer sex education by outreach workers and visits by outreach workers, sex worker peer educators, men who have sex with men peer educators, and/or mobile HIV‐care clinics at venues.

Characteristics of the population in this study were defined by responses to bio‐behavioural survey questions provided by venue participants, hereafter: “participants.” Condom availability was measured by an affirmative response to “If you wanted a condom, would it be easy for you to get one?”, “In the past six months, has an outreach worker such as a peer educator given you a condom?”, or “Do you have a condom with you now and can you show it to me [the interviewer]?”. Effective condom use was measured from a series of four questions asking about the participation in vaginal sex in the prior year, the use of condom at last vaginal sex, the participation in anal sex in the prior year and the use of condom at last anal sex, where “I have never had penis to vagina/anal sex” was a response option.

Venues were classified as having a high percentage of persons living with HIV if prevalence among participants at the venue was in the top 33% of HIV prevalence estimates across all study venues. Similarly, venues were classified as having a high percentage of persons with unsuppressed viral load if the prevalence of unsuppressed viral load at the venue was in the top 33% of the prevalence distribution among study venues. HIV status was defined by the result from a rapid HIV test, except among those who refused the test or had a missing test result; in such cases, participants who reported a positive test result within the prior year were classified as living with HIV. Viral suppression was determined through analysis of dried blood spots and was defined as a viral load measurement under 1,000 copies/mL (see details in Data S1).

### 2.5 Statistical analysis

Two types of data were analysed: venue‐level data and participant‐level data. Venues were weighted to represent the distribution of characteristics across all venues at the selected cross‐border areas. Participant‐level data were weighted to represent the distribution of behaviours and other characteristics that would be observed among a random sample of individuals at venues at the selected cross‐border areas. The participant‐level data weights used to generate weighted estimates for behaviours, characteristics and viral suppression are described in the Data S1.

Applying weights and adjusting standard errors to account for the complex survey design, we estimated the distribution of venue characteristics, including type of venue and availability of HIV prevention services. At the participant level, using person‐level weights and accounting for survey design, we estimated the distribution of demographic characteristics among populations found at venues in these cross‐border areas and the distribution of HIV prevention behaviours, such as condom use, access to condoms and lubricants, and receipt of information about HIV prevention.

We estimated the weighted mean number of prevention services and the proportion of participants reporting high‐risk behaviours for venues where a high percentage of persons living with HIV or unsuppressed HIV were found. We generated prevention cascades for condom availability, and effective use using weighted percentages for men, women, female sex workers and men who have sex with men. Among uninfected persons visiting venues, HIV testing history was categorized by ever been tested for HIV, testing in the prior year and receipt of test result. Finally, we generated maps of cross‐border areas depicting the weighted percentage of venues with condoms available where visitors had difficulty accessing condoms and the percentage of venues visited by education outreach where visitors had low effective use of condoms. Analyses were conducted using SAS 9.4 software (SAS institute, Cary, NC) and R (version 3.5.4).

## 3 RESULTS

### 3.1 Study sample

Of 1161 venues sampled for venue verification, 883 were found operational with an informant willing to answer questions about the venue. Overall, 45.3% of venues were bars or pubs (Table 1). According to venue informants, 94.8% of venues were visited by members of at least one key or priority population, men or women visited the venue to pay for or sell sex at 55.0% of the venues, and among the venues where women and men were having sex, 71.9% (95% CI: 67.3, 76.4) had condoms available at the venue.

**Table 1:** Characteristics of venues in the East Africa Cross‐Border Integrated Health Study, 2016‐2017

	All venues (n = 883)		
	Count	Weighted %	95% CI
Venue type			
Bar/pub/restaurant	407	45.3	41.7, 48.9
Commercial venue^a^	74	9.4	7.2, 11.7
Hotel/guest house/lodge	266	29.9	26.6, 33.1
Nightclub/disco/brothel	22	2.4	1.3, 3.6
Outside venue^b^	39	4.5	3.0, 6.1
Transportation hub^c^	13	1.6	0.7, 2.6
Other	62	6.8	5.0, 8.6
Populations visiting venue			
Fisher folk	386	44.7	41.1, 48.3
Truck drivers	609	69.7	66.5, 73.0
Young women	282	34.0	30.5, 37.5
Homeless people	297	35.2	31.7, 38.7
Injection drug users	51	6.2	4.4, 8.0
Years in operation			
Less than 1 year	117	12.7	10.4, 15.1
More than 1 year	742	87.3	84.9, 89.6
Sale of alcohol	576	63.8	60.3, 67.3
Sex at venue	438	49.1	45.6, 52.6
Persons looking to pay for or sell sex at venue	471	55.0	51.4, 58.7
Female sex worker lives at venue	155	17.9	15.0, 20.7

a Commercial venues included markets, hair salons, shops, cinemas, recreation and game centres and schools;

b Outdoor venues included beaches, parks, construction sites and streets;

c Transportation hubs included truck stops and lorry/railway stations. CI, confidence interval.

Among the 883 venues, 452 were sampled for the bio‐behavioural interviews. At sampled venues, 11,410 individuals participated in the survey. The average age of participants was 30 years, 66.2% were male, 75.3% were employed and 46.1% had at least some secondary education (Table 2). Among women at venues, 21.3% (95% CI: 19.3, 23.2) reported that they had ever experienced physical violence and 12.4% (95% CI: 10.7, 14.1) had ever been forced to have sex. Among participants who were not living with HIV, 89.7% had previously received an HIV test, however, 20.9% of participants reported their last test was more than one year prior to the survey, not including the test offered at the time of the survey (Data S1).

**Table 2:** Demographics of participants in the East Africa Cross‐Border Integrated Health Study, 2016‐2017

	All participants (n = 11,410)		
	Unweighted mean/frequency	Weighted %	95% CI
Age – Mean (min, max)	30.2	30.4 (15, 85)	30.0, 30.7
Female	4175	33.8	32.1, 35.4
Pregnant^a^	244	8.2	6.8, 9.6
Education			
Less than primary school	2326	21.4	19.8, 23.1
Primary school	3827	32.5	30.8, 34.1
Some secondary or more	5241	46.1	44.1, 48.2
Employment			
Full, partial or informal	8511	75.3	73.4, 77.3
Not employed	2793	24.7	22.7, 26.6
Key populations			
Young women	1653	13.0	11.9, 14.1
Female sex workers	655	5.3	4.7, 6.0
Men who have sex with men	92	0.8	0.6, 1.0
Persons who inject drugs	55	0.6	0.4, 0.8
Fisher folk	1279	9.9	7.7, 12.1
Truck drivers	192	1.9	1.3, 2.4

a Weighted percentage of women who were pregnant at the time of interview among all women. Ci, confidence interval.

### 3.2 The condom cascade

**Figure 1:**
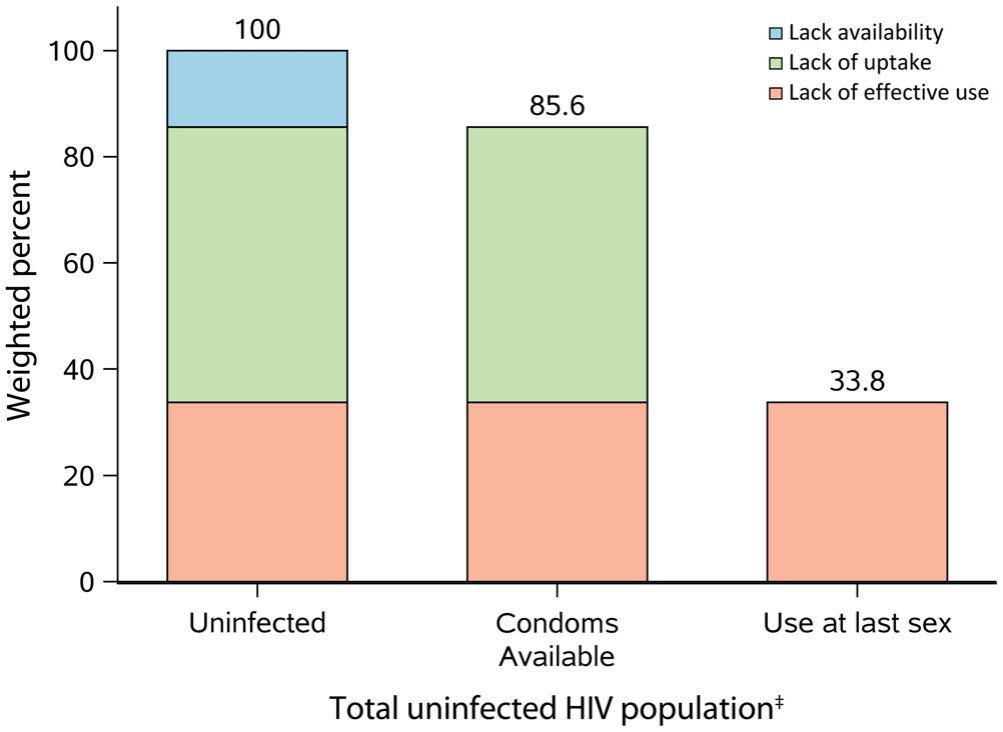
HIV intervention‐centric prevention cascade among an uninfected HIV population in the East Africa Cross‐Border Integrated Health Study, 2016‐2017 ^a^Color categories were generated using existing HIV prevention cascade frameworks [17]. ^b^Uninfected are those who had sex in the prior year and were not infected with HIV.

**Figure 2:**
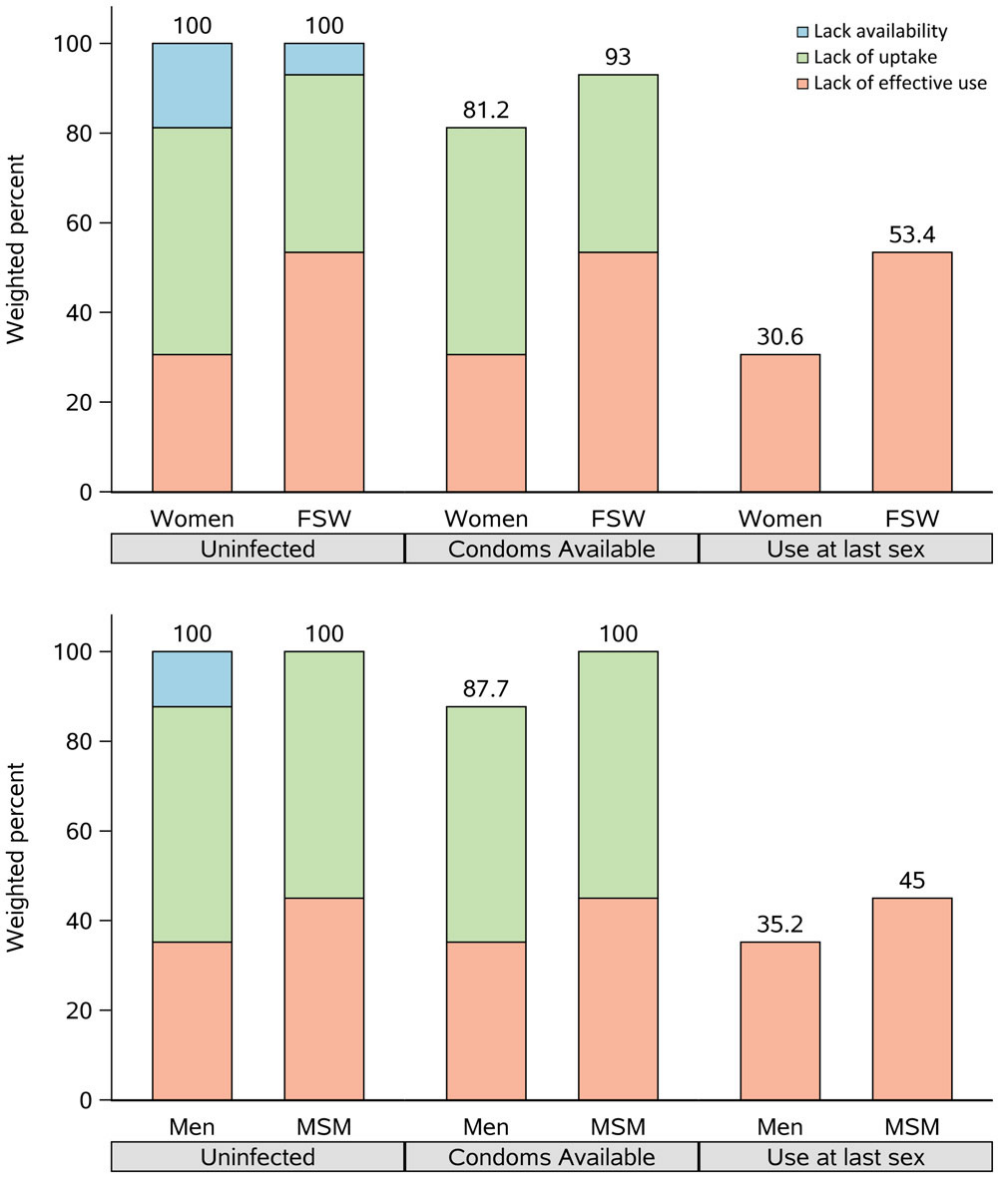
HIV intervention‐centric prevention cascade in select populations in the East Africa Cross‐Border Integrated Health Study, 2016‐2017^a^. ^a^Colour categories were generated using existing HIV prevention cascade frameworks [17]. ^b^Uninfected are those who had sex in the prior year and were not infected with HIV.

The HIV prevention cascades for condoms demonstrate that the principal obstacle to condom use was lack of effective use (Figure 1). Among the 85.6% of participants who reported being able to obtain a condom, 60.5% reported not using a condom at their last sexual encounter (51.8% of the total uninfected population). However, a subset of participants reported difficulty accessing condoms (14.4%), with women (18.8%) more likely to report lack of access as a barrier to condom use than men (12.3%, Figure 2). Female sex workers at venues were more likely to report having access to and using condoms than other participant populations. Most men who have sex with men at venues reported access to condoms but low effective use.

### 3.3 Access to prevention services

Among participants, 92.6% of women and 92.4% of men reported having received information about HIV/AIDS either at the venue, on the radio, or from a health worker in the year prior to their interview (Table 3). Among participants who came to venues to find a sex partner (n = 1,040), 11.4% (95% CI: 7.6, 15.2) carried a condom with them. While 54.0% (95% CI: 47.7, 60.2) of those engaged in anal sex in the prior 12 months (n = 183) reported using a condom at their last anal sexual encounter, only 36.7% (95% CI: 34.9, 38.6) of those participating in vaginal sex (n = 9275) in the prior 12 months reported using a condom at last vaginal sex. Among participants reporting both anal and vaginal sex in the prior 12 months (n = 161), 44.0% reported consistent use of a condom at last anal and vaginal sexual encounter. Among participants reporting receipt of any HIV information and vaginal sex in the prior 12 months, 37.4% reported using condoms at last vaginal sexual encounter.

**Table 3:** Access to prevention services among participants in the East Africa Cross‐Border Integrated Health Study, 2016‐2017

	Women (n = 4175)			Men (n = 7235)		
	Unweighted mean/ frequency	Weighted %	95% CI	Unweighted mean/ frequency	Weighted %	95% CI
Condom access and use						
Feel it is easy to get condom	2960	71.1	68.7, 73.5	5823	80.1	78.7, 81.6
Given condom by outreach worker in prior six months	1650	37.5	34.6, 40.4	3017	41.6	38.8, 44.4
In possession of a condom	192	4.3	3.3, 5.4	323	4.1	3.4, 4.9
Used condom at last anal sex (among those who had anal sex in the prior 12 months, **n = 183**)	44	48.9	41.9, 55.8	32	43.9	36.0, 51.8
Used condom at last vaginal sex (among those who had vaginal sex in the prior 12 months, **n = 9275**)	1173	35.7	32.9, 38.6	2200	37.3	35.2, 39.3
Other prevention services						
HIV testing in prior 12 months	3138	74.0	71.9, 76.1	4909	67.3	65.5, 69.2
Feel it is easy to get sexual lubricants	201	4.6	3.0, 6.1	305	4.0	3.1, 4.9
Circumcised (among men)	‐	‐	‐	5506	77.0	75.1, 78.9
Received information about HIV/AIDS at the venue in the prior 12 months	2189	50.3	47.5, 53.2	3597	48.7	45.6, 51.8
Received information about HIV/AIDS on the radio in the prior 12 months	3652	86.2	84.7, 87.8	6426	88.4	87.0, 89.9
Received information about HIV/AIDS from health worker in the prior 12 months	3003	69.1	66.1, 72.1	4927	66.9	64.5, 69.3
Received information about HIV/AIDS from any source in the prior 12 months	3842	92.6	91.4, 93.7	6676	92.4	91.4, 93.5

### 3.4 Alignment of prevention services and high‐risk venues

Venues visited by high percentages of people living with HIV, overall or unsuppressed, were not substantially more likely to have condoms available, according to venue informants than all venues combined (Table 4, 54.8% and 58.3% vs. 51.8%). Informants at venues in outside areas and transportation hubs, like railway stations and truck stops, reported an average of at least three prevention services across all cross‐border areas (Table 5). The average number of prevention services offered per venue ranged from 0.1 to 3.4 at each cross‐border area with notable differences in available services across border areas in Malaba, Katuna/Gatuna and Mutukula (Data S1). The most commonly reported prevention services included distribution or sale of male condoms and availability of HIV testing.

**Table 4:** Venue characteristics and HIV prevention services available at venues with high percentages (top tertile) of persons living with HIV and persons who are not virally suppressed in cross‐border areas in the East Africa Cross‐Border Integrated Health Study, 2016‐2017

	Venues with high % of visitors living with HIV (n = 90)^a^		Venues with high % of virally unsuppressed visitors (n = 28)^a^		All Venues (n = 883)	
	Weighted %^b^	95% CI	Weighted %^b^	95% CI	Weighted %^b^	95% CI
Venue characteristic (%)						
Venue type						
Bar/pub/restaurant	49.1	38.0, 60.2	33.3	13.9, 52.6	45.3	41.7, 48.9
Commercial spot^c^	3.8	0.0, 7.6	9.3	0.0, 20.9	9.4	7.2, 11.7
Hotel/guest house/lodge	29.8	19.4, 40.1	25.9	10.2, 41.7	29.9	26.6, 33.1
Nightclub/disco/brothel	3.2	0.0, 6.9	‐	‐	2.4	1.3, 3.6
Outside areas^d^	10.0	3.4, 16.7	19.2	3.1, 35.4	4.5	3.0, 6.1
Transportation hub^e^	0.8	0.0, 2.4	2.8	0.0, 8.5	1.7	0.7, 2.6
Other	3.3	0.0, 6.6	9.5	0.0, 24.1	6.8	5.0, 8.6
Condoms available^f^	54.8	44.3, 65.2	58.3	36.1, 80.5	51.8	48.2, 55.3
Alcohol sold	71.0	60.6, 81.4	53.7	31.6, 75.9	63.8	60.3, 67.3
Sex takes place on‐site	58.8	47.9, 69.8	59.9	39.8, 80.1	49.1	45.6, 52.6
Sex work at venue	75.9	65.9, 85.9	53.7	34.9, 72.6	55.0	51.4, 58.7
Female sex workers live at venue	25.0	15.5, 34.5	22.2	5.8, 38.7	17.9	15.0, 20.7
Mean number of prevention services						
Overall	2.15	1.58, 2.72	2.26	1.26, 3.25	1.79	1.63, 1.96
At venues where alcohol is available and/or sex takes place on‐site	2.34	1.72, 2.96	2.54	1.32, 3.76	2.02	1.83, 2.20

a In the venues with high proportions of persons living with HIV, 8.3% to 40.7% of the persons visiting that venue live with HIV. In the venues with high proportions of persons living with unsuppressed HIV, 100% of the persons with HIV visiting that venue live with unsuppressed HIV;

b Data weighted and standard errors adjusted to account for survey design;

c Commercial spots included markets, hair salons, shops, cinemas, recreation and game centres and schools;

d Outside areas include beaches, parks, sex worker streets and construction sites

Transportation hubs included truck stops and lorry/railway stations;

Condoms available for free or for sale. CI, confidence interval. CI, confidence interval.

**Table 5:** Average number of HIV prevention services available by venue type in cross‐border areas in the East Africa Cross‐Border Integrated Health Study, 2016‐2017

	All Areas (n = 883)		
	Number of venues	Weighted mean	95% CI
Venue type			
Bar/pub/restaurant	407	1.65	1.41, 1.89
Commercial spot^a^	74	1.47	0.98, 1.95
Hotel/guest house/lodge	266	1.77	1.51, 2.04
Nightclub/disco/brothel	22	2.10	1.26, 2.94
Outside area^b^	39	3.03	1.84, 4.22
Transportation hub^c^	13	3.67	1.70, 5.64
Other	62	1.92	1.24, 2.60

a Commercial spots included markets, hair salons, shops, cinemas, recreation and game centres and schools;

b Outdoor area included beaches, parks, construction sites and streets;

c Transportation hubs included truck stops and lorry/railway stations.

### 3.5 Disparities in availability and use of services

**Figure 3:**
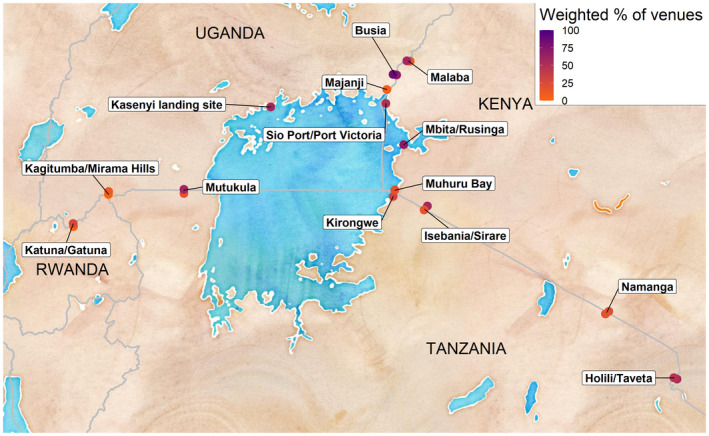
Map of cross‐border areas in East Africa included in the East Africa Cross‐Border Integrated Health Study (2016‐2017) with weighted percentages of venues with free male/female condoms available in the prior six months among all venues visited by uninfected persons having difficulty accessing condoms.

**Figure 4:**
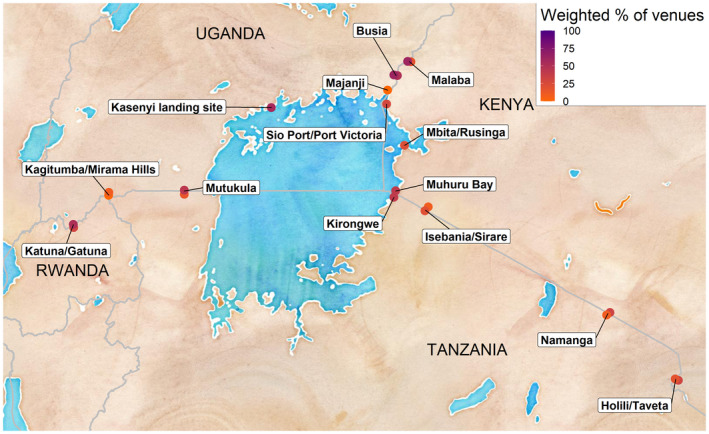
Map of cross‐border areas in East Africa included in the East Africa Cross‐Border Integrated Health Study (2016‐2017) with weighted percentages of venues visited by education outreach among all venues visited by uninfected persons not using condoms.

In 12 of the 22 cross‐border areas, informants reported that free male or female condoms were available in the prior six months at less than 33.0% of the venues where participants reported difficulty accessing condoms at the venue (Figure 3). For 17 of the 22 cross‐border areas, informants reported that, in the past six months, education outreach had visited less than 50.0% of the venues where participants reported low effective use of condoms (Figure 4). Many cross‐border areas had large disparities in condom availability and use across the international border; in these areas, one side of the border may have many venues with both a high need for condoms (i.e. many participants who reported low access or effective use of condoms) and few services to address this gap (i.e. many informants reported no free condoms available and no education outreach at venues) despite high reported levels of access to/effective use of condoms and outreach activities at venues just across the border.

## 4 DISCUSSION

The CBIHS identified important successes and opportunities for improving HIV prevention programming at cross‐border areas in East Africa. We described a study population that was composed mostly of young men, suggesting that outreach to venues in cross‐border areas may be a suitable strategy to reach men with HIV testing and other prevention services. Our analysis used novel HIV prevention cascades to visually represent data on HIV prevention intervention delivery. With this prevention cascade framework, we found that effective use at last sex was low despite generally high access to condoms. Our approach provides an illustrative example for how prevention cascades can be applied to guide assessment of other HIV prevention commodities, like PrEP and personal lubricants [17, 18, 19, 20]. Lastly, we observed important differences in the availability of prevention services by venue type and within the same cross‐border areas, suggesting opportunities for more granular targeting of venue‐based prevention services and transnational coordination of HIV prevention programming.

Application of the cascade framework provides programme planners with a standardized way to visualize barriers to targeted HIV prevention delivery. The cascade format enables quick visualization of important opportunities (or the lack thereof) to prioritize and provide HIV prevention interventions. In our study, the application of the cascade framework identified adherence to condom use as a major barrier to overall effective use in cross‐border areas. The cascade framework also creates a standardized way to compare efforts across different populations at risk, such as between men and men who have sex with men and between women and female sex workers as was done in our study. Based on such comparisons, it appears additional work must be done to reach men and women at venues who are not men who have sex with men or female sex workers, but who may nonetheless be at higher risk for HIV acquisition than people in the general population. Finally, while we focused on condoms for the prevention cascade in our study, the framework can extend to other prevention commodities, like PrEP [26].

The above programmatic applications notwithstanding, the prevention cascade has unique challenges compared to the traditional HIV care and treatment cascade, and has received some criticism for its limitations [27]. Many of these limitations have been identified previously in other applications of the prevention cascade framework to real‐world data [17, 18, 19, 26, 28]. For example estimating the percentages contained in each step of a prevention cascade requires collecting more information directly from participants than the traditional HIV care and treatment cascade. Because the CBIHS was not designed explicitly to produce an HIV prevention cascade, we did not capture all elements of the cascade for all prevention interventions, which limited our ability to describe the cascade for services like HIV testing and counselling and voluntary medical male circumcision. An additional challenge for applying prevention cascades is unpacking local or national influences on the availability and uptake of prevention services. For example the low effective use of condoms in our study population could have been driven by the approval of PrEP for use in East African countries; however, this is an unlikely explanation since PrEP availability was nascent at selected study sites during the time of the CBIHS.

We described small differences by sex in condom use at last vaginal sex, consistent with prior reports from the region of differences in effective condom use by sex [29]. These observations may be explained partly by structural barriers to condom use, such as economic and gender inequalities and intimate partner violence (IPV), which disproportionately affect women and can hinder women’s ability to negotiate for consistent condom use. It has been shown that women who have experienced IPV are 1.5 times more likely to acquire HIV than women who have not experienced IPV [30, 31, 32]. A significant percentage of women visiting venues in cross‐border areas experienced physical violence or violence during sex, which might explain the lower condom use observed. Other structural factors have been shown to impact condom use, including poverty, alcohol use before sex and policing practices [33, 34]. To improve condom and other HIV prevention commodity uptake and efficacy at cross‐border areas, HIV prevention programmes must carefully screen for structural barriers and offer one or more integrated mitigation services, such as IPV support services, peer navigation, substance use disorder treatment and income generating activities, among others [35].

HIV prevention programmes should deploy structural interventions in combination with evidence‐informed biomedical and behavioural prevention interventions tailored to the needs of the community and the local context [2]. HIV prevention programmes may capitalize on opportunities to create synergies between combination interventions. For example receipt of HIV/AIDS education was high in our study population, and this can serve as an important starting point for promoting HIV testing services, condom adherence, or new prevention methods like PrEP. Similarly, existing HIV testing and peer educator services at venues can be leveraged to improve condom uptake through condoms provision, education on HIV risk during pre‐ and post‐test counselling, and HIV behaviour change communication to promote condom use.

This study identified other opportunities for improving HIV prevention programmes. The preponderance of men found at cross‐border venues suggests possibilities to implement targeted HIV case‐finding strategies that engage men with novel HIV testing modalities, such as index testing and HIV self‐testing, and linkage to treatment or prevention as appropriate. Similarly, we described how bars, pubs and restaurants had a higher proportion of patrons living with HIV, but a lower average number of available HIV prevention services than other types of venues. This heterogeneity in availability of services across venue type suggests that current HIV prevention programming may not be sufficiently tailored to site‐level differences in the epidemic at cross‐border areas. Targeting HIV prevention interventions to the people and places that need them most can be a more efficient and impactful way to deliver HIV services [36].

This study has several limitations. First, the small sample size for some key and priority populations in our study, such as men who have sex with men and persons who inject drugs, limit inferences about the prevention needs and access to services of these groups in selected cross‐border areas. Second, the analytic weights used do not account for bias introduced by informative refusals or participants leaving the venue when they learn that a survey is being conducted. Moreover, self‐reported responses to the bio‐behavioural survey have potential to introduce recall bias and social desirability bias into respondent data. Finally, the CBIHS did not examine the effects of social gender norms, income inequality, human rights violations on access to and uptake of, HIV prevention services and future studies should collect data on these structural factors.

To improve HIV prevention programming in East African cross‐border areas, future studies should examine whether recent investments in transnational coordination and service delivery since the time of the CBIHS have overcome the barriers to prevention uptake and adherence documented here [1, 2, 5, 30]. Implementation science research, including empirically supported frameworks and measures, may clarify the extent to which evidence‐informed HIV prevention interventions are being routinely deployed in combination to reach key and priority populations in cross‐border areas, as well as the reach, adoption, sustainability and effectiveness of such efforts. Recently, several interventions have been proposed and/or implemented in cross‐border areas, such as introducing cross‐border health units, liberalizing access to health services across borders, and creating harmonized, cross‐border HIV care and treatment protocols. Acquiring knowledge about the effectiveness, sustainability and scalability of these interventions will be vital to improving HIV prevention in these areas. Also, we need more data on the context and infrastructure required to successfully implement interventions to improve HIV knowledge and condom availability in cross‐border areas and populations. New knowledge on effective strategies to adapt existing behaviour change communication for cross‐border populations is needed to better encourage effective use of condoms and other HIV prevention commodities. Finally, further research is needed to refine the prevention cascade framework to be maximally relevant to real‐world HIV prevention settings, and to capture the dynamic nature of HIV risk behaviour and client preferences for various combinations of HIV prevention services, including PrEP [21, 37].

## 5 CONCLUSIONS

There remain critical opportunities to improve HIV prevention for key and mobile populations at venues in East African cross‐border areas, specifically to increase availability and use of condoms and other primary HIV prevention interventions. Delivering effective HIV prevention in this setting requires adapting programming to fit the local HIV epidemic, including tailoring services to address granular differences in service availability and uptake by demographic and risk behaviour profile, venue type and geographical location. Applying the prevention cascade framework helps improve HIV prevention interventions by standardizing visualization of barriers to prevention delivery, quickly highlighting gaps, comparing efforts across different populations at risk and identifying priorities for future HIV prevention efforts. Harnessing this unique and important data source to examine access to and gaps in prevention services can be a useful and complementary public health tool to protect the populations in cross‐border areas.

## COMPETING INTERESTS

One author (PA) is employed by the funding agency. The other authors declare no conflicts of interest.

## AUTHORS’ CONTRIBUTIONS

AV performed the analysis and wrote the manuscript. PA and FS were involved in designing and conducting the study and revised the manuscript. GEM and MM were involved in study design, overseeing data collection, analysis, and revision of the manuscript. MH was involved in the revision of the manuscript. SW was involved in study design and revised the manuscript. JKE designed the study, oversaw data collection, participated in data analysis and revised the manuscript.

## Supporting information


**Data S1.** Additional results and details on methodology and statistical analysis
