# Supplementary material for: Access to HIV prevention services in East African cross‐border areas: a 2016‐2017 cross‐sectional bio‐behavioural study
Source: J Int AIDS Soc. 2020 Jun 30;23(Suppl 3):e25523. doi: 10.1002/jia2.25523 (PMC7325514; doi:10.1002/jia2.25523)
Supplement: Supplementary file 1 — Data S1. Additional results and details on methodology and statistical analysis [file JIA2-23-e25523-s001.DOC]

**Supplement for “Access to HIV prevention services in East African cross-border areas: a 2016-2017 cross-sectional bio-behavioral study”**

**HIV testing among uninfected participants in the East Africa Cross-Border Integrated Health Study, 2016-2017**

**
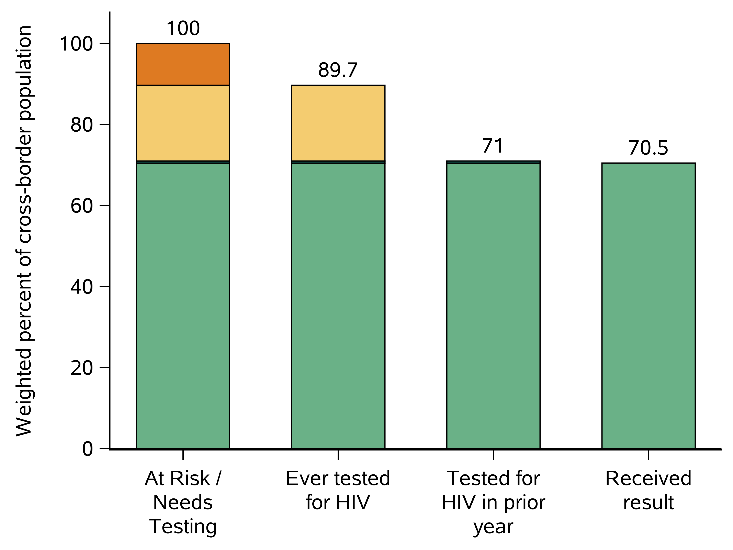

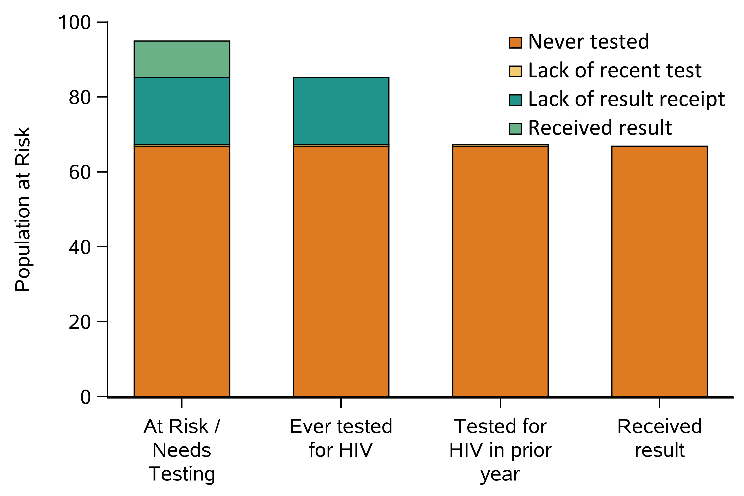
**

**Average number of preventive services by cross-border area in the East Africa Cross-Border Integrated Health Study, 2016-2017**

|  | **All Venues (n=883)** | | |  |
| --- | --- | --- | --- | --- |
|  | **Count** | **Weighted mean**  **(95% CI)** | **National reference** | **HIV prevalence** |
| **Border Area** |  |  |  |  |
| Malaba, Kenya | 26 | 1.68 (0.85, 2.51) | 2.07 (1.74, 2.40) | 3.3 (1.4, 5.3) |
| Malaba, Uganda | 40 | 2.95 (2.18, 3.72) | 2.46 (2.14, 2.79) | 4.8 (2.9, 6.6) |
| Busia, Kenya | 29 | 3.40 (2.19, 4.62) | 2.07 (1.74, 2.40) | 3.6 (0.4, 6.8) |
| Busia, Uganda | 61 | 3.41 (2.66, 4.16) | 2.46 (2.14, 2.79) | 3.0 (1.4, 4.6) |
| Katuna/Gatuna, Rwanda | 26 | 1.25 (0.61, 1.89) | 0.95 (0.42, 1.48) | 1.2 (0.1, 2.2) |
| Katuna/Gatuna, Uganda | 41 | 2.26 (1.36, 3.16) | 2.46 (2.14, 2.79) | 4.4 (2.7, 6.1) |
| Taveta, Kenya | 48 | 2.05 (1.44, 2.66) | 2.07 (1.74, 2.40) | 1.5 (0.0, 3.1) |
| Holili, Tanzania | 28 | 1.50 (0.87, 2.14) | 1.25 (0.97, 1.53) | 3.2 (1.4, 5.1) |
| Sio Port/Port Victoria/Majanji, Kenya | 45 | 1.37 (0.80, 1.95) | 2.07 (1.74, 2.40) | 3.8 (2.1, 5.5) |
| Sio Port/Port Victoria/Majanji, Uganda | 30 | 0.89 (0.41, 1.38) | 2.46 (2.14, 2.79) | 6.3 (3.2, 9.3) |
| Isebania/Sirare, Kenya | 30 | 1.13 (0.70, 1.57) | 2.07 (1.74, 2.40) | 4.1 (1.8, 6.4) |
| Isebania/Sirare, Tanzania | 49 | 0.96 (0.54, 1.38) | 1.25 (0.97, 1.53) | 4.7 (2.5, 6.9) |
| Namanga, Kenya | 20 | 1.20 (0.21, 2.19) | 2.07 (1.74, 2.40) | 1.3 (0.0, 2.9) |
| Namanga, Tanzania | 59 | 0.92 (0.50, 1.33) | 1.25 (0.97, 1.53) | 3.9 (2.4, 5.4) |
| Kagitumba, Rwanda | 18 | 0.11 (0.0, 0.27) | 0.95 (0.42, 1.48) | 2.2 (0.3, 4.2) |
| Mirama Hills, Uganda | 51 | 0.56 (0.24, 0.88) | 2.46 (2.14, 2.79) | 5.9 (3.7, 8.1) |
| Mbita landing site and Rusinga Island, Kenya | 69 | 1.70 (1.28, 2.12) | 2.07 (1.74, 2.40) | 6.8 (5.2, 8.5) |
| Kasenyi landing site, Uganda | 62 | 2.40 (1.77, 3.04) | 2.46 (2.14, 2.79) | 4.5 (3.1, 5.8) |
| Muhuru Bay, Kenya | 30 | 3.06 (1.65, 4.46) | 2.07 (1.74, 2.40) | 7.6 (5.4, 9.8) |
| Kirongwe, Tanzania | 36 | 1.54 (0.69, 2.40) | 1.25 (0.97, 1.53) | 3.1 (1.5, 4.7) |
| Mutukula, Tanzania | 45 | 1.07 (0.66, 1.47) | 1.25 (0.97, 1.53) | 6.0 (3.6, 8.4) |
| Mutukula, Uganda | 40 | 2.76 (1.90, 3.62) | 2.46 (2.14, 2.79) | 7.3 (4.8, 9.8) |

PLACE methods:

The method requires three main steps: enumeration of venues where populations of interest socialize, verification and characterization of venues, and bio-behavioral interviews at venues. For the CBIHS, populations of interest included female sex workers, men who have sex with men, people who inject drugs, fisher folk, truck drivers, and mobile populations.

At each area, local officials were consulted to identify types of community members likely to be knowledgeable about local venues and the populations of interest. Data collectors were assigned targets for these community member types and were dispatched to busy public areas. They approached potential informants in accordance with the targets, and each informant was asked to describe up to 10 venues in the cross-border area where people were likely to find new sexual partners.

Venues were de-duplicated from lists, and unique venues were sorted by reported presence of key populations and onsite sexual activity, and frequency of report. Venues were assigned to higher, medium, and lower priority strata based on the sort order. At cross-border areas where 100 or fewer venues were listed, data collectors visited all venues for verification. At areas where more than 100 venues were listed, a stratified random sample of venues was visited for verification. At these areas, sampling probability was highest for venues in the higher priority stratum and lowest for venues in the lower priority stratum. Data collectors attempted to locate the venues selected for verification, and at those which were found and operational, they identified a knowledgeable person who could answer questions about the venue.

Data collectors visited a stratified random sub-sample of the verified venues for bio-behavioral interviews. At these venues, data collectors interviewed male and female patrons and workers, with targets set in proportion to the number present at each venue. Data collectors used hand-held tablets to guide patrons and venue workers through a questionnaire about personal characteristics including sociodemographic information, health-seeking and sexual behaviors, and exposure to HIV prevention and treatment intervention. All participants were offered an onsite HIV test, though participants who did not consent to the test were still invited to participate in the interview. Viral load measurements were communicated back to the facility from which the local HIV counseling and testing staff responsible for counselling and testing were associated. Respondents were given a card with an ID code and facility name so they could obtain their viral load results.

Viral load testing:

Viral load tests for Kenyan and Ugandan sites were completed in their respective countries. Viral load tests for Tanzanian and Rwandan sites were completed in Uganda at Uganda Virus Research Institute (UVRI) laboratories. UVRI laboratories used Roche COBAS AmpliPrep/TaqMan 48 FVE (free virus elution protocol) to process the dried blood spots.

How the sampling weights were generated:

Sampling weights were used to weight the study sample to the population of interest: people visiting venues at the cross-border areas. The weights were designed to account for variations in sampling probabilities across survey participants and venues. For example, individuals who were recruited at a venue that had a relatively low a probability of selection were up-weighted to represent additional people who could have been recruited from similar venues had we visited all venues in the cross-border site.

Sampling weights were applied to venue-level and individual data. Unweighted, venue-level data reflect the distribution of venue characteristics in a sample of operational venues at the cross-border areas. Once weights are applied, results reflect the distribution of characteristics that would have been observed if all venues in a cross-border area had been visited or if a simple random sample of venues in the cross-border area had been assessed rather than a stratified random sample.

Sampling weights were also applied to the biobehavioral survey data. Unweighted, biobehavioral survey data reflect the distribution of population characteristics in a sample of male workers, female workers, male patrons, and female patrons present at a sample of venues at the time of data collection. With weights applied, the data reflect the distribution of population characteristics that would have been observed if a simple random sample of people at venues in cross-border sites has been taken, rather than a stratified random sample of people at a stratified random sample of venues.

Taken together, weighted data depict the characteristics of venues and populations at venues across the 12 cross-border areas included in the study.

How unsuppression weights were generated:

For measures related to HIV testing, data were weighted by the product of the survey sampling weights and HIV test refusal weights. The HIV test refusal weight was estimated as the marginal probability of refusing the HIV test divided by the probability of refusing the test conditional on informative covariates. Probabilities were estimated using multivariable logistic regression, with the conditional probability of refusing the test modeled as a function of a respondent’s sex, age group, cross-border area where interviewed, employment at the place of interview, and timing and reported result of last HIV test. Categorical covariates were modeled using indicator variables. For viral suppression results, missing viral load weights were similarly estimated among the women, dividing the marginal probability of a missing viral load by the conditional probability, conditional on the woman’s age group, cross-border area where interviewed, and employment at the place of interview. In estimating viral suppression results, data were weighted by the product of three component weights: the survey sampling weight, the HIV test refusal weight, and the missing viral load weight.

Population size estimates

Cross-border areas of all sizes were included in this study ranging from Kirongwe, Tanzania with a population of 4,771 (according to the National Bureau of Statistics Tanzania 2012 Census) to Busia, Uganda with a population of 54,798 (according to the Uganda Bureau of Statistics 2014 Census).

Venue informant and respondent perspectives of access to condoms

Among persons visiting venues where male and female condoms are distributed for free, 17.9% (95% CI: 15.9, 19.9) of respondents did not feel it was easy to get access to a condom.

**Operationalization of indicators**

| **Measure** | **Questions and values** |
| --- | --- |
| Average numbers of prevention services | Affirmative response by venue informant of occurrence within the past 6 months:  Has [select HIV prevention activity] occurred at this spot within the past 6 months, longer than 6 months ago, or never occurred here? HIV prevention activities included: distribution of free male condoms, distribution of free female condoms, distribution of free sexual lubricant, condoms for sale at spot, persons tested at spot for HIV, safer sex education by outreach workers, visits by outreach workers, visits by sex worker peer educators, visits by MSM peer educators, visits by a mobile clinic, needle exchange program, and male circumcision programs. |
| Education outreach | Affirmative response by venue informant:  Has [select outreach] occurred at this spot within the past 6 months, longer than 6 months or it has never occurred here. Outreach includes: safer sex education by outreach workers, visits by outreach workers, sex worker peer educators, men who have sex with men peer educations, and/or mobile HIV-care clinics at venues |
| Condom availability | Affirmative response by biobehavioral survey participant (venue visitor) to any of these questions:  “If you wanted a condom, would it be easy for you to get one?”, “In the past 6 months, has an outreach worker such as a peer educator given you a condom?”, or “Do you have a condom with you now and can you show it to me [the interviewer]?” |
| Effective condom use | Affirmative response to either by biobehavioral survey participant (venue visitor):  Participated in vaginal sex in the prior year and use of condom at last vaginal sex Participated in anal sex in the prior year and use of condom at last anal sex |

**HIV intervention-centric prevention cascade in select populations in the East Africa Cross-Border Integrated Health Study, 2016-2017**


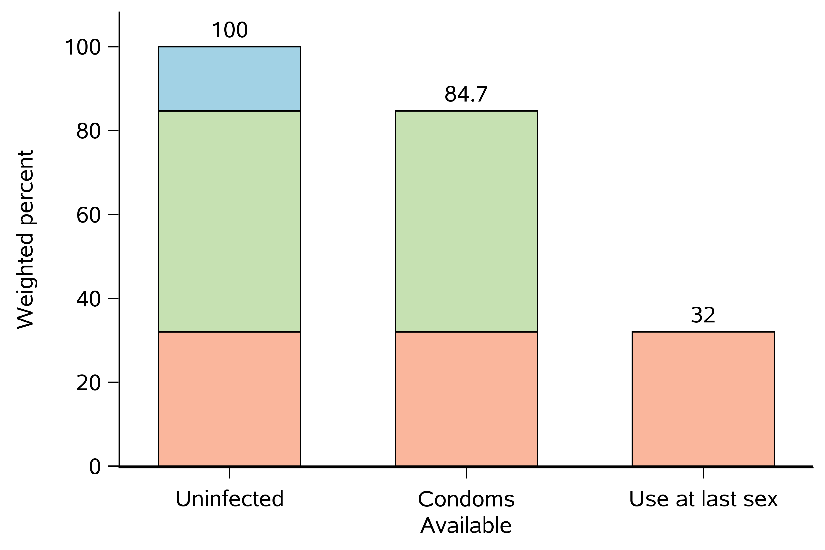

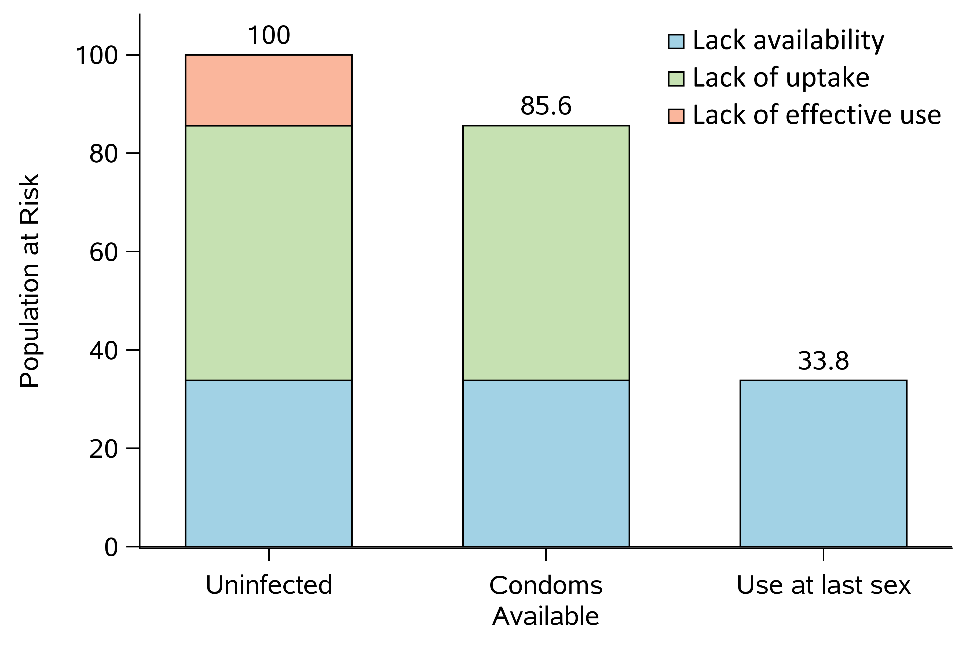

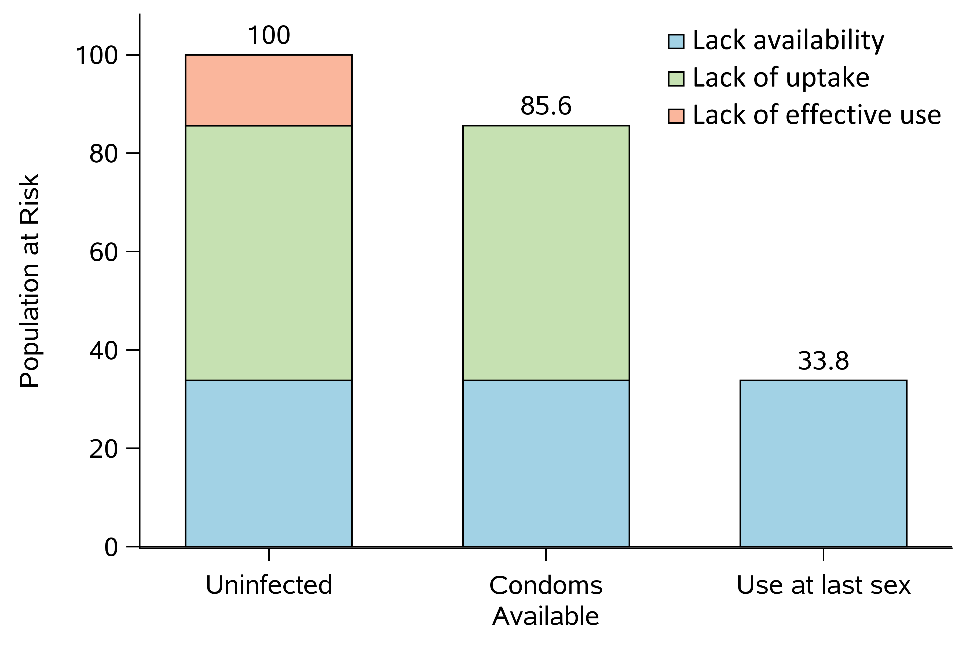

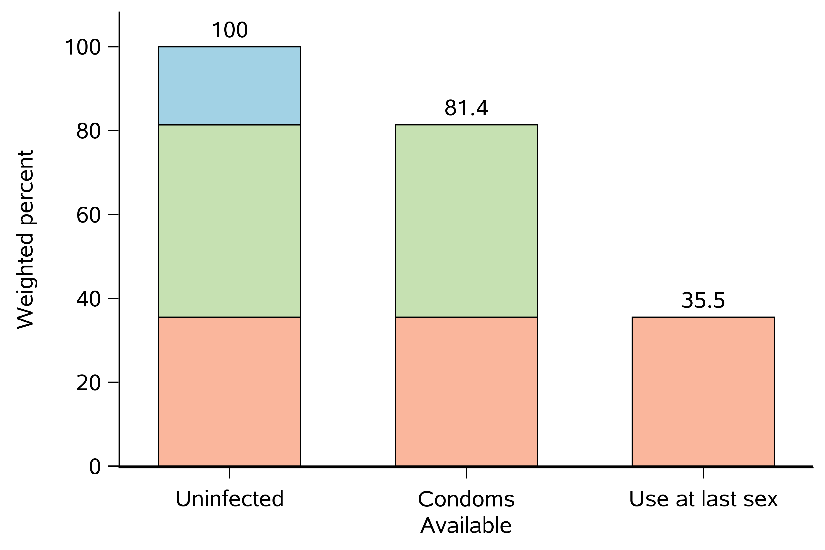
**Uninfected Young Women Uninfected Fisher Folk**

† Color categories were generated using existing HIV prevention cascade frameworks

‡ Uninfected are those who had sex in the prior year and were not infected with HIV

**Access to prevention services of women and female sex workers in the East Africa Cross-Border Integrated Health Study, 2016-2017**

|  | **Women (n=4175)** | | | **Female Sex Workers (n=655)** | | |
| --- | --- | --- | --- | --- | --- | --- |
|  | **Unweighted mean/**  **frequency** | **Weighted %** | **95% CI** | **Unweighted mean/**  **frequency** | **Weighted %** | **95% CI** |
| **Condom access and use** |  |  |  |  |  |  |
| Feel it is easy to get condom | 2960 | 71.1 | 68.7, 73.5 | 559 | 87.3 | 83.9, 90.8 |
| Given condom by outreach worker in prior 6 months | 1650 | 37.5 | 34.6, 40.4 | 347 | 51.8 | 45.2, 58.5 |
| In possession of a condom | 192 | 4.3 | 3.3, 5.4 | 97 | 13.3 | 9.4, 17.3 |
| Used condom at last anal sex (among those who had anal sex in the prior 12 months, **n=183**) | 44 | 48.9 | 41.9, 55.8 | 14 | 41.7 | 33.4, 50 |
| Used condom at last vaginal sex (among those who had vaginal sex in the prior 12 months, **n=9275**) | 1173 | 35.7 | 32.9, 38.6 | 340 | 58.7 | 52.5, 64.9 |
| **Other prevention services** |  |  |  |  |  |  |
| HIV testing in prior 12 months | 3138 | 74.0 | 71.9, 76.1 | 535 | 79.2 | 74.6, 83.8 |
| Feels it is easy to get sexual lubricants | 201 | 4.6 | 3.0, 6.1 | 53 | 8.6 | 3.3, 13.9 |
| Received information about HIV/AIDS from outreach worker at the venue in the prior 12 months | 2189 | 50.3 | 47.5, 53.2 | 377 | 54.6 | 48.6, 60.6 |
| Received information about HIV/AIDS on the radio in the prior 12 months | 3652 | 86.2 | 84.7, 87.8 | 572 | 86.4 | 82.5, 90.3 |
| Received information about HIV/AIDS from health worker in the prior 12 months | 3003 | 69.1 | 66.1, 72.1 | 485 | 72.8 | 68.1, 77.6 |
| Received information about HIV/AIDS from any source | 3842 | 92.6 | 91.4, 93.7 | 612 | 92.9 | 90.4, 95.4 |

**Access to prevention services of men and men who have sex with men in the East Africa Cross-Border Integrated Health Study, 2016-2017**

|  | **Men (n=7235)** | | | **Men who have sex with men (n=183)** | | |
| --- | --- | --- | --- | --- | --- | --- |
|  | **Unweighted mean/**  **frequency** | **Weighted %** | **95% CI** | **Unweighted mean/**  **frequency** | **Weighted %** | **95% CI** |
| **Condom access and use** |  |  |  |  |  |  |
| Feel it is easy to get condom | 5823 | 80.1 | 78.7, 81.6 | 85 | 96.6 | 94.8, 98.5 |
| Given condom by outreach worker in prior 6 months | 3017 | 41.6 | 38.8, 44.4 | 48 | 51.8 | 38.7, 64.9 |
| In possession of a condom | 323 | 4.1 | 3.4, 4.9 | 5 | 2.3 | 0.2, 4.4 |
| Used condom at last anal sex (among those who had anal sex in the prior 12 months, **n=183**) | 32 | 43.9 | 36.0, 51.8 | 4 | 68.4 | 2.3, 100 |
| Used condom at last vaginal sex (among those who had vaginal sex in the prior 12 months, **n=9275**) | 2200 | 37.3 | 35.2, 39.3 | 26 | 45.8 | 33.1, 58.5 |
| **Other prevention services** |  |  |  |  |  |  |
| HIV testing in prior 12 months | 4909 | 67.3 | 65.5, 69.2 | 61 | 67.3 | 57.3, 77.3 |
| Feels it is easy to get sexual lubricants | 305 | 4.0 | 3.1, 4.9 | 4 | 2.7 | 0, 5.5 |
| Circumcised (among men) | 5506 | 77.0 | 75.1, 78.9 | 65 | 66.9 | 57.3, 76.6 |
| Received information about HIV/AIDS from outreach worker at the venue in the prior 12 months | 3597 | 48.7 | 45.6, 51.8 | 56 | 61.9 | 45.5, 78.2 |
| Received information about HIV/AIDS on the radio in the prior 12 months | 6426 | 88.4 | 87.0, 89.9 | 71 | 77.7 | 70.2, 85.3 |
| Received information about HIV/AIDS from health worker in the prior 12 months | 4927 | 66.9 | 64.5, 69.3 | 55 | 67.1 | 51.1, 83.1 |
| Received information about HIV/AIDS from any source | 6676 | 92.4 | 91.4, 93.5 | 78 | 86.5 | 74.3, 98.6 |
